# Retrospective cause analysis of troponin I elevation in non-CAD patients: Special emphasis on sepsis

**DOI:** 10.1097/MD.0000000000008027

**Published:** 2017-09-15

**Authors:** Chien-Wen Yang, Huijun Li, Lisa Thomas, Manuel Ramos, Po-Hong Liu, Thomas Roe, Ravinder Valadri, Michael C. Kiel, Vincent Yi-Fong Su, Qi Shi

**Affiliations:** a Department of Internal Medicine, The Wright Center for Graduate Medical Education (WCGME), Scranton; b Hematology & Oncology Associates of Northeast Pennsylvania; c Department of Epidemiology, Harvard T.H. Chan School of Public Health; d Commonwealth Health Physician Network Great Valley Cardiology; e Department of Family Medicine, WCGME; f Department of Science, Marywood University, Scranton, PA; g Faculty of Medicine, National Yang-Ming University, Taipei, Taiwan.

**Keywords:** cause analysis, non-CAD, retrospective, sepsis, troponin I elevation

## Abstract

**Background:**

Troponin I is one of the most commonly tested biochemical markers in the emergency room (ER) and in the hospital setting. Besides coronary artery disease (CAD), demand ischemia with underlying tachycardia, anemia, hypertensive emergency, congestive heart failure, kidney disease, sepsis, and pulmonary embolism have also been reported to cause troponin I elevations. Few reports have excluded patients with CAD, and no study has summarized the proportion of these factors relative to an increased troponin I level.

**Methods:**

The aim of this retrospective study was to investigate the level of contribution of causative factors in troponin I elevation. Charts of patients tested for troponin I during an ER visit or during hospitalization were collected. Patients with known CAD, abnormal stress tests, cardiac catheterizations, or discharge without an adequate cardiac evaluation were excluded. Logistic regression was used to identify predictors of elevated troponin I levels.

**Results:**

A total of 586 patients were investigated in this study. Age, hemoglobin (Hb), heart rate (HR), glomerularfiltration rate, atrial fibrillation, congestive heart failure (CHF), and sepsis were significant predictors of elevated troponin I by analysis in univariate logistic regression (all *P* < .001). In multivariate logistic regression, sepsis, CHF, age, Hb, and HR were independent predictors of troponin I (all *P* < .01). A simple clinical scoring system was generated with 1 score on patients with age ≥ 60, Hb < 10 g/dL, and HR ≥ 100 beats per minute (bpm). The prevalence of elevated troponin I was 4%, 16%, 38%, and 50% for patients with scores of 0, 1, 2, and 3, respectively. In patients without sepsis and CHF, the chances of elevated troponin I were 2%, 11%, 28%, and 43%.

**Conclusions:**

Sepsis was found to be the strongest independent cause of elevated troponin I levels in non-CAD patients. The scoring system composed of age, hemoglobin (Hb), and heart rate (HR) can assist clinical evaluation of elevated troponin I test in non-CAD patients.

## 1 Introduction

Troponin I is one of the most commonly tested biochemical markers in the emergency room (ER) and hospital setting. Elevated troponin I is diagnostic for myocardial injury.^[1]^ Coronary artery disease (CAD) has been well known to cause the elevation of troponin I.^[2]^ Other than CAD, increased oxygen demand of cardiomyocytes or decreased myocardial blood flow can also cause elevated troponin I, also known as type II myocardial infarction. The causes of demand ischemia have been reported as tachycardia, anemia, hypertensive emergency, congestive heart failure, kidney disease, sepsis, and pulmonary embolism (PE).^[3,4]^ Individual analysis of each underlying diagnosis has been shown in multiple studies. Troponin I elevation has been documented in sepsis patients without CAD on autopsy.^[5]^ Multiple reports identified troponin I elevations in patients with congestive heart failure (CHF).^[6,7]^ It has been reported that the heart rate can be a primary dependent factor in patients with paroxysmal supraventricular tachycardia (PSVT) without CAD.^[8]^ Elevated troponin I levels were documented in patients with atrial fibrillation (Afib).^[9]^ Anemia in sickle cell crisis and acute gastrointestinal bleeding have been identified as causes of troponin I elevation ^[10,11]^ as was hypertensive crisis.^[12]^ Patients with chronic kidney diseases tended to have baseline troponin I elevation.^[13]^ Half of patients diagnosed with PE had similar laboratory findings.^[14]^ However, there were only a few reports that excluded patients with CAD, and no study has summarized the proportion of these factors. The aim of this retrospective study was designed to investigate alternate diagnoses other than CAD that could contribute to troponin I elevations.

## 2 Methods

### 2.1 Patients

Patients tested for troponin I in a local community hospital in Scranton, PA, USA, between November 1, 2016, and December 15, 2016, were included. Exclusion criteria included patients with known CAD or coronary etiologies, positive stress test or evidence of CAD based on cardiac catheterization results during hospitalization, and patients who were discharged (death or transfer) without proper evaluation. The study was approved by the Institutional Review Board.

### 2.2 Data collections

Analytic data were collected including age, sex, hemoglobin level (Hb), heart rate (HR), systolic blood pressure (SBP), and glomerular filtration rate (GFR) by the Cockcroft Gault equation and underlying conditions, including CHF, sepsis, PE, and Afib.^[15]^ The assay of troponin I utilized was the high sensitivity troponin I assay. Elevation of troponin I was defined according to the parameter of the hospital and was equal or greater than 0.03 ng/mL. If there were multiple troponin level measured during one hospital visit, only the highest one was documented. Among multiple Hb level, HR, SBP records, only the closest one to the troponin I test was documented. The clinical data were collected only once for the patients with multiple admissions.

### 2.3 Statistical analysis

Normality assumption was not met, so the authors employed the Mann–Whitney *U* test to examine continuous outcome between patients with and without elevated troponin I levels. The chi-square test and 2-tailed Fisher exact test were used to evaluate categorical data. The association between elevated troponin I level and clinical variables by binary logistic regression was analyzed. Potential predictors of elevated troponin I included age, gender, hemoglobin level, heart rate, systolic blood pressure, glomerular filtration rate, presence of atrial fibrillation, congestive heart failure, pulmonary embolism, and sepsis. These were comprehensively included in the univariate logistic regression. Factors with a *P* value less than 0.1 in the univariate model were introduced in to the multivariate logistic regression to estimate the adjusted odds ratio (OR) and 95% confidence interval (CI) by forward selection. A simple clinical score was created to predict elevated troponin I based on the results of multivariate logistic regression. The discriminatory ability of the clinical score was evaluated using concordance statistics (c-statistics). Statistical analysis was conducted with SPSS for Windows version 20.0 (IBM, NY). Statistical significance was set at a 2-tailed *P* value less than 0.05.

## 3 Result

**Figure 1:**
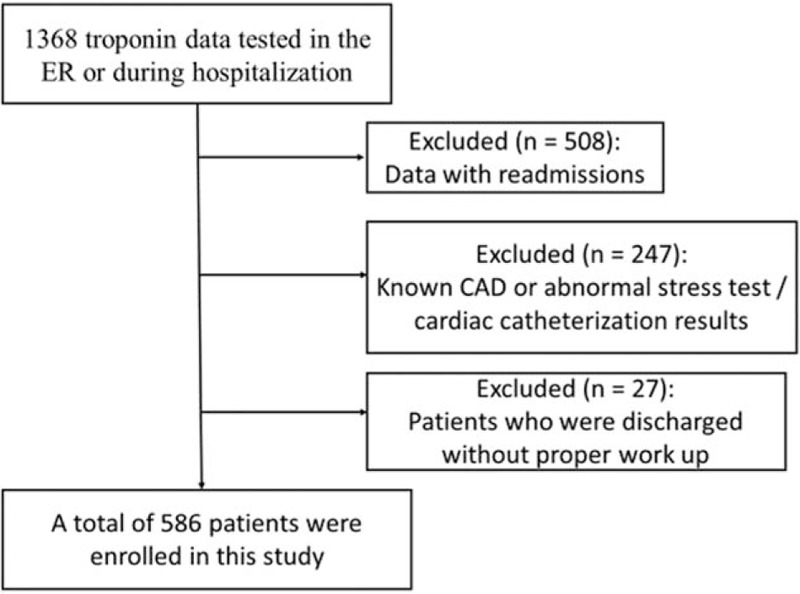
Flow diagram summarizing the process of enrollment. CAD = coronary artery disease, ER = emergency room.

A total of 1368 cases were tested for troponin I during an ER visit or hospitalization. Five hundred and eight patients were omitted due to readmission. Two hundred and forty-seven patients had known CAD or abnormal stress test/cardiac catheterization results during admission. Twenty-seven patients were discharged (died or being transferred) without proper evaluation. A total of 586 patients were included in this study (Fig. 1).

Baseline characters of the cohort are summarized in Table 1. The mean age was 65 years old with 265 patients (45%) being male. Among the 586 patients, 97 patients (17%) were found to have elevated troponin I levels, 19 patients (3%) were admitted with a diagnosis of sepsis, 54 patients (9%) had a history of CHF, 87 patients (15%) had Afib, 7 patients (1%) were diagnosed with pulmonary embolism, and 7 patients (1%) were being treated with dialysis. A total of 47 patients (8%) were found to have hemoglobin levels less than 10, 115 patients (20%) were found to have an HR greater than 100 beats per minute, and SBP greater than 180 mm Hg were recorded in 16 patients (3%). Echocardiogram (echo) reports were analyzed in 47 patients with a history of CHF, and 20 of them had reduced ejection fractions. Total mean ejection fraction per available echo reports in CHF patients was 44%. In total, 6 out of 47 patients with Hb less than 10 g/dL had developed acute anemia secondary to hemodilution or acute blood loss.

**Table 1:**
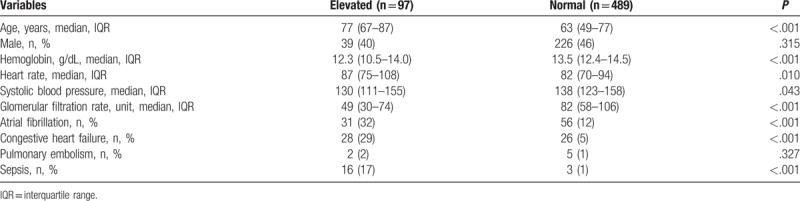
Baseline demographics among patients with and without elevated troponin I.

Patients with elevated troponin I differed from patients without elevated troponin I (Table 1). Patients with elevated troponin I were older, had lower HB, SBP, and GFR (all *P* < .05). Patients with elevated troponin I also had higher HR and were more likely to have Afib, CHF, and sepsis (all *P* < .05). In univariate logistic regression, age, HB, HR, GFR, Afib, CHF, and sepsis were associated with higher odds of having elevated troponin I (all *P* < .05, Table 2). When introduced into multivariate logistic regression, age, HB, HR, CHF, and sepsis remained independent predictors of having elevated troponin I. When the analysis was restricted to patients without sepsis or CHF (n = 516), age, HB, and HR were consistently independent predictors that contributed to elevated troponin I.

**Table 2:**
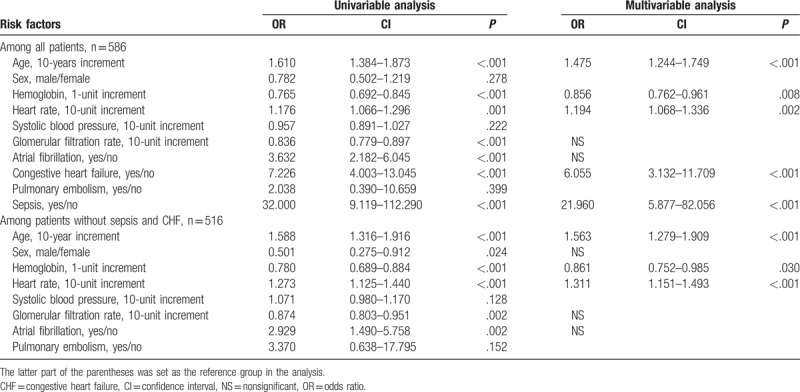
Univariate and multivariate logistic regression analyses for elevated troponin I among all patients and among patients without sepsis and congestive heart failure.

**Figure 2:**
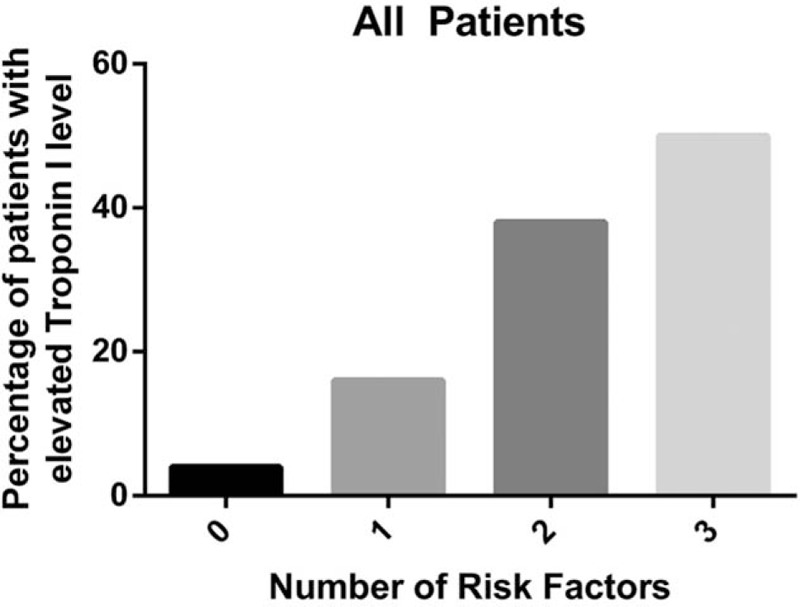
Proportion of patients with an elevated troponin I value according to the number of risk factors.

**Figure 3:**
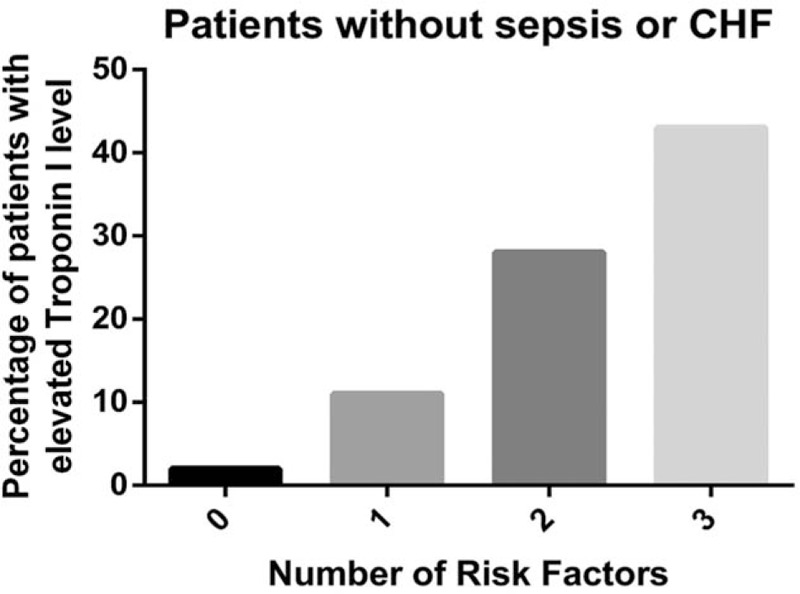
Proportion of patients with an elevated troponin I value according to the number of risk factors among patients without sepsis or congestive heart failure (CHF). CHF = congestive heart failure.

A simple clinical score for predicting elevated troponin I was created based on the results from multivariate logistic regression (Table 3, Figs. 2 and 3). The authors dichotomized the significant predictors (age, HB, and HR) so that the score was clinically feasible. The prevalence of elevated troponin I was 4%, 16%, 38%, and 50% for patients with scores of 0, 1, 2, and 3, respectively. In patients without sepsis and CHF, the chances of elevated troponin I were 2%, 11%, 28%, and 43%. The score was associated with moderate discriminatory ability (c-statistics: 0.712, 95% confidence interval [CI]: 0.657–0.767). When the analysis was restricted to patients without sepsis or CHF, the score again showed moderate discriminatory ability (c-statistics: 0.726, 95% CI: 0.658–0.794).

**Table 3:**
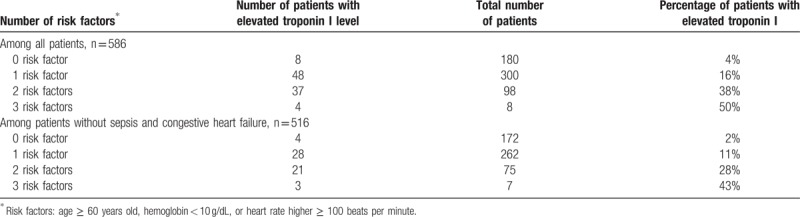
Simple clinical score for predicting abnormal troponin I levels among all patients and among patients without sepsis and congestive heart failure.

## 4 Discussion

The authors identified sepsis as the strongest independent factor of troponin I elevation in non-CAD patients in this study. In previous reports, sepsis caused increased cytokines secretion, catecholamine surge, and tissue metabolic demand. Increased oxygen demand in peripheral hypoxic tissue can further increase the cardiomyocytes workload and cause relative insufficient coronary blood flow.^[16,17]^ The forensic report also demonstrated evidence that sepsis caused elevated troponin I without underlying CAD.^[5]^ Though troponin T has been reported as a prognostic factor in sepsis patients,^[18]^ troponin I (which is the more common subtype in United States) on day 1 of hospitalization was reported to have no prognostic value in sepsis patients.^[19]^ It is not an uncommon practice for septic patients to have troponin I levels checked in the ER on admission. The authors conclude that checking troponin I levels in septic patients, especially without underlying CAD, has to be done discriminately to try to curtail and additional unnecessary testing.

CHF was also identified as an independent cause of elevated troponin I. Congestive heart failure is known to cause sporadic areas of cardiomyocytes death, abnormal structures of the remaining cardiomyocytes, and ultimately progression to fibrosis. Thus, troponin I elevation can be caused by the cell necrosis process.^[20]^ There are multiple articles that describe elevated troponin I in CHF patients.^[6,7,20]^ In this study, the authors identified CHF as an independent cause of elevated troponin I level in non-CAD patients. However, CHF can also be a complication of acute coronary syndrome.^[21]^ Thus, definitive decisions concerning the monitoring and interpretation of elevated troponin I levels will depend on clinical judgment during an ER visit or hospitalization.

Age was also noted in this study to correlate with increased troponin I levels. It has been reported that age greater than 60 years old is a significant factor that can affect a troponin I level.^[22]^ Thus, the authors concluded that age related differences should be taken into consideration when interpreting the troponin I level.

Heart rate was also an independent factor that affected the troponin I level. Heart rate has been well known to be a risk factor that causes increased oxygen demand. This can lead to relative insufficient coronary blood flow.^[23]^ It has been reported that heart rate can be primary dependent factor in patients with supraventricular tachycardia without CAD.^[8]^ This study reconfirmed that HR has a strong impact on troponin I levels in non-CAD patients.

Anemia was another diagnosis that was associated with elevated troponin I in this study. Anemia can cause a hyperdynamic status by increasing heart rate and contractility, which can further increase the oxygen demand of cardiomyocytes which then results in insufficient coronary blood flow.^[23,24]^ Abruptly increased cardiac index in patients with acute anemia has been demonstrated in a previous article.^[25]^ Anemia in sickle cell crisis and acute gastrointestinal bleeding were also reported to be associated with troponin I elevation.^[10,11]^ In this study, anemia was confirmed to be a single independent factor for troponin I elevation. However, there was no significant difference between acute and chronic anemia regarding troponin I levels. There were only 6 patients who were diagnosed with acute anemia included in this study. Due to the number of cases, this cohort will need to be further studied.

The authors generated a simple clinical scoring system with 1 score given to patients with Hb < 10 g/dL, age ≥ 60 and HR ≥ 100 beats per minute. This was applied to patients without known or clinical suspicion of CAD. Given that sepsis and CHF were the 2 strongest independent factors, the scoring system was initially done on all patients with a second analysis done on those patients excluding sepsis and CHF. With the lowest score, the chance of the patient having an elevated troponin I was 4% versus 2%; with the highest score, the chance of the patient having an elevated troponin I level was 50% versus 43%. The score showed moderate discriminatory ability and provided a tool that could be applied in clinical practice to try to identify subsets of patients who might have elevated troponin I levels due to the factors identified above and who do not generate a high clinical suspicion of CAD.

In this report, SBP showed significant results in the Mann–Whitney test but showed insignificant results in logistic regression. The data were further analyzed. It was identified that within SBP 140 to 180 mm Hg, the percentage of abnormal troponin I was around 7% but increased to 16% to 29% in the rest of the SBP intervals. The nonlinear correlation explained the statistic difference. Hypertensive crisis has been well described as a cause of cardiomyocytes ischemia. The underlying pathophysiology could be due to impaired blood pumping from the heart due to increased afterload.^[26]^ However, hypotension was also listed as a factor for elevated troponin I.^[3]^ The underlying pathophysiology may be due to reduced coronary perfusion during a hypotensive episode.^[23]^ Further investigation is needed regarding hypertension and hypotension as causes of elevated troponin I levels.

Atrial fibrillation (Afib) was shown to have a significant effect on troponin I value in chi-Square and univariate logistic regression in this study. However, Afib failed to show independent significant impact in multivariate logistic regression. In this study, 87 patients were diagnosed with Afib. Out of the 87, 79 patients were greater than age 60, and 20 patients had HR greater than 100 beats per minute (bpm). This implies that age and HR can be confounding factors in patient diagnosed with Afib. Parwani et al analyzed 354 patients with Afib and concluded troponin I release can be induced by Afib. However, the study included CAD patients, and there was no control group of patients without Afib.^[27]^ Thus, the association between Afib and elevated troponin I levels needs to be further investigated.

In this study, GFR showed no significant effect on troponin I. The method used to calculate the GFR was based on the Cockcroft Gault equation. None of the patients had quantified standard GFR urine test. Age was a numerator on the Cockcroft Gault equation and 193 out of 217 patients with GFR < 60 mL/min/1.73 m^2^ are age greater than 60 years old. This implies that age can also be a confounding factor in GFR in this study. It has been reported that troponin I elevation is associated with chronic kidney disease and decrease GFR.^[12]^ Ziebig et al enrolled 24 patients after heart surgery with measurement of both serum and urine troponin I and classified the patients based on urine GFR. This suggested that impaired kidney function affected the troponin I removal. However, 8 out of 24 patients had myocardial infarction as a complication of CAD in the study.^[28]^ Thus, a further prospective study with quantified GFR levels in non-CAD patients may provide more information.

It has been reported that half of the patients with PE have elevated troponin I.^[14]^ In this study, PE associated with increased troponin I levels was not statistically significant. This may be due to the insufficient sample size (only 7 patients with PE in this study).

There is 1 recently published study that revealed that sex, age, and SBP are strong factors of elevated troponin I in healthy adults with mean age 37 years old.^[29]^ However, both SBP and sex failed to show statistical significance in this study. Based on real world clinical practice, the mean age of the patient in this study was age 65 years and many of the patients had comorbid ailments as listed.

Current research regarding troponin I focuses on single factors as causative etiologies of elevated levels and rarely excludes CAD patients. In this study, patients with underlying CAD were excluded. This gave the authors an independent assessment of the other factors that led to increased troponin I levels.

There were several limitations in this study. This was a retrospective study in a single center. Not all the patients had definite diagnostic tests such as cardiac catheterization, computed tomography angiogram, or magnetic resonance imaging to rule out CAD. However, all the medical records were reviewed and the patients enrolled were evaluated by hospitalists and cardiologists based on clinical judgment of symptoms and risk stratifications. All the patients with known CAD, abnormal stress test/cardiac catheterization results and patients without proper cardiac evaluation were excluded. Before doing this, the highest troponin I level was greater than 80 ng/mL; after excluding the CAD patients, the highest troponin I level decreased to 1.79 ng/mL. This implies that the patients left after omitting CAD patients had milder cardiomyocytes damage. A larger prospective study is needed for further investigation. Finally, although the score showed moderate discriminatory ability within the cohort, external validation is still needed to confirm the results.

## 5 Conclusion

Troponin I is one of the commonly tested biochemical markers reflecting underlying cardiomyocytes damage. In this study, sepsis was identified as the strongest independent cause of elevated troponin I levels in non-CAD patients. Thus, the authors concluded that troponin I levels should be discriminately checked on septic patients, especially without underlying CAD. The scoring system composed of age, Hb, HR provided an additional tool to assist in predict potential troponin I abnormalities.
